# Spiny and Non-spiny Parvalbumin-Positive Hippocampal Interneurons Show Different Plastic Properties

**DOI:** 10.1016/j.celrep.2019.05.098

**Published:** 2019-06-25

**Authors:** Angelica Foggetti, Gilda Baccini, Philipp Arnold, Thomas Schiffelholz, Peer Wulff

**Affiliations:** 1 Institute of Physiology, Christian-Albrechts-University Kiel, 24098 Kiel, Germany; 2 Anatomical Institute, Christian-Albrechts-University Kiel, 24118 Kiel, Germany; 3 Department of Psychiatry and Psychotherapy, Christian-Albrechts-University Kiel, 24105 Kiel, Germany

**Keywords:** dendritic spines, interneurons, plasticity, parvalbumin, perineuronal net, pnn, dentate gyrus, enriched environment, re-wiring, cluster

## Abstract

Dendritic spines control synaptic transmission and plasticity by augmenting post-synaptic potentials and providing biochemical compartmentalization. In principal cells, spines cover the dendritic tree at high densities, receive the overwhelming majority of excitatory inputs, and undergo experience-dependent structural re-organization. Although GABAergic interneurons have long been considered to be devoid of spines, a number of studies have reported the sparse existence of spines in interneurons. However, little is known about their organization or function at the cellular and network level. Here, we show that a subset of hippocampal parvalbumin-positive interneurons forms numerous dendritic spines with highly variable densities and input-selective organization. These spines form in areas with reduced perineuronal net sheathing, predispose for plastic changes in protein expression, and show input-specific re-organization after behavioral experience.

## Highlights

• A subset of PV+ interneurons (PVIs) in the dentate gyrus shows high spine density
• Spiny PVIs have less perineuronal net wrapping and show enhanced plasticity
• Spines on PVIs show clustering and input-dependent plasticity
• Spiny and aspiny dendrites are differently rewired in experience-induced plasticity

## Graphical Abstract

**Figure:**
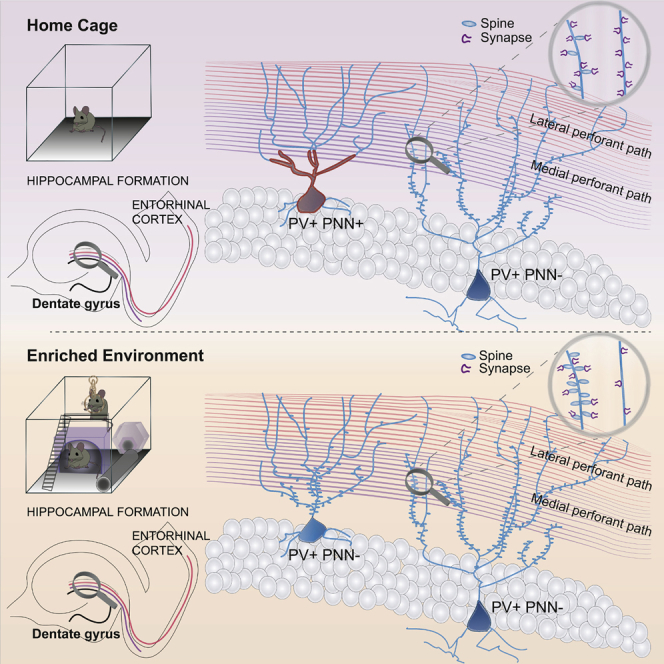

## Teaser

Dendritic spines on excitatory principal cells are critical sites of learning-induced plasticity. Foggetti et al. report that a subset of parvalbumin-positive interneurons in the dentate gyrus carries high densities of clustered dendritic spines, which predispose for plastic changes in gene expression and affect experience-dependent rewiring.

## Introduction

Experience-based changes of synaptic strength and neuronal connectivity form the basis of learning and memory (Bliss and Lomo, 1973, Hebb, 1949, Holtmaat and Svoboda, 2009, Yuste and Bonhoeffer, 2001). Dendritic spines, which in principal cells (PCs) receive the vast majority of excitatory inputs, are thought to be critical sites of plasticity (Holtmaat and Svoboda, 2009, Yuste and Bonhoeffer, 2001). These dendritic protrusions minimize interference between excitatory inputs by electrical isolation and by compartmentalization of post-synaptic calcium transients and molecular signaling cascades, which in turn control the strength of synaptic transmission (Holtmaat and Svoboda, 2009). In cortical PCs, structural changes of dendritic spines, including the generation of new and elimination of existing spines, underlie long-term changes of synaptic strength, connectivity, and memory formation (Hayashi-Takagi et al., 2015, Yuste and Bonhoeffer, 2001). Parvalbumin-expressing GABAergic interneurons (PVIs) comprise mainly basket and axo-axonic cells. These neurons target the perisomatic region of postsynaptic cells and are critical regulators of PC activity (Murray et al., 2011). Due to their rapid action potential firing, phase locked to neuronal network oscillations, and their ion channel and receptor expression profile, they have been suggested to form a rigid interconnected network geared toward rapid signaling and oscillatory entrainment of PC ensembles (Hu et al., 2014, Klausberger et al., 2003). In line with this concept, PVIs have been reported to be largely devoid of dendritic spines (Freund and Buzsáki, 1996, Gulyás et al., 1999), but see Kawaguchi et al. (2006) and Sancho and Bloodgood (2018), a property which may aid in rapid signal propagation and fast input to output conversion (Hu et al., 2014). However, recent reports have shown behavior-dependent changes in protein expression in hippocampal PVIs (Donato et al., 2015), remodeling of their axonal branches (Pieraut et al., 2014) and functional plasticity at excitatory inputs targeting PVIs (Hainmueller et al., 2014), suggesting that these neurons participate in experience-induced network plasticity. Inspired by several studies on the mostly sparse occurrence of spines on different types of cortical interneurons (Guirado et al., 2014, Kawaguchi et al., 2006, Keck et al., 2011, McBain et al., 1994, Sancho and Bloodgood, 2018, Scheuss and Bonhoeffer, 2014), we assessed the existence and organization of dendritic spines in PVIs of the hippocampal formation in adult mice. We show that a fraction of PVIs in the dentate gyrus (DG) but not the cornu ammonis (CA) areas 1 and 3 carry high densities of dendritic spines. These spines form in areas with weakly developed perineuronal nets (PNNs), display non-homogeneous input-dependent distributions, predispose for plastic changes, and show input-specific re-organization after behavioral experience.

## Results

### A Fraction of PVIs in DG but Not CA Areas Carries High Densities of Dendritic Spines

**Figure 1:**
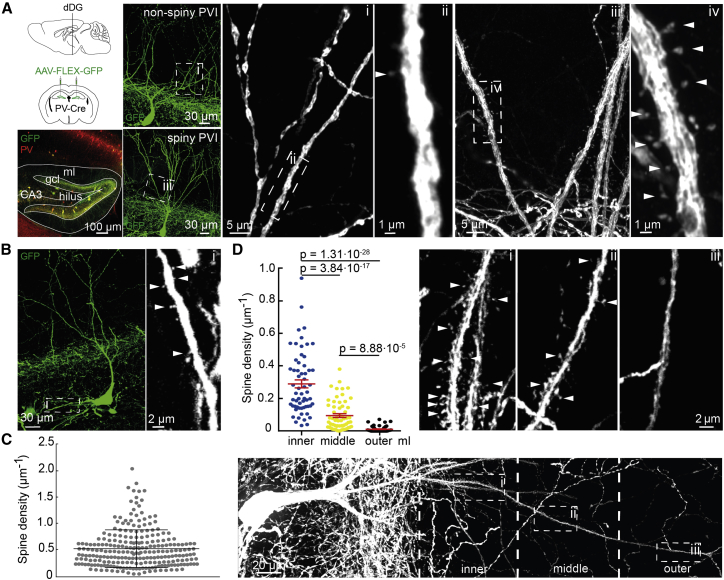
A Subpopulation of PVIs in the dDG Expresses Variable and Input-Specific Densities of Dendritic Spines (A) AAV-FLEX-GFP was injected into the dDG of PV-Cre mice. Co-localization of GFP (green) and PV (red) was confirmed by immunohistochemistry. Example images of transduced non-spiny (top) and spiny (bottom) apical dendrites of PVIs are shown alongside confocal gray-scale magnifications of boxed areas. Roman numbers link boxed areas to matching magnifications. Arrowheads indicate examples of spines. (B) Example of spines on basal PVI dendrites with a gray scale magnification of the boxed area. (C) Spine densities of individual apical dendritic segments show high variability (n = 248 segments, 37 neurons, 3 mice; bars are mean ± SD). (D) Apical spine densities decrease from inner to outer ml (n = 60 neurons, 6 mice; bars are mean ± SEM). Right, images i, ii, and iii show higher magnifications of a typical spiny PVI dendrite crossing the ml sublayers (bottom).

To visualize dendrites of PVIs throughout the dorsal hippocampus, we initially labeled PVIs with tdTomato in PV-Cre::Ai9 mice (∼80% of tdTomato-positive cells were immuno-positive for PV; Figure S1). Whereas dendrites of PVIs in CA1 and CA3 were largely non-spiny or sparsely spiny (Hu et al., 2014), we detected many tdTomato-positive dendrites of PV-immunoreactive neurons with high spine densities in the dorsal DG (dDG) (Figure S1). To reduce mislabeling caused by developmental expression of Cre recombinase and to reveal the organization of spines in single cells, we subsequently labeled PVIs by injecting an adeno-associated virus (AAV) carrying the GFP reading frame inverted in a flip-excision cassette (AAV-FLEX-GFP) into different hippocampal sub-regions of adult PV-Cre mice (Figure 1A). This approach permitted sparse and faithful labeling of PVIs (∼95% of GFP-positive cells were PV-positive; n = 318 neurons, 3 mice; Figure 1A) and confirmed that PVIs in the CA1 and CA3 areas were mostly non-spiny (Figure S2). In the dDG, however, 60 of 102 GFP-labeled PVIs (58.8%) had spiny apical dendrites. Quantification of spines using NeuronStudio software (Rodríguez et al., 2008) revealed densities ranging from 0.02 to 0.43 spines μm^−1^ when measured over the entire dendritic tree (mean density, 0.13 ± 0.01 spines μm^−1^). The other 42 PVIs had smooth apical dendrites with <0.02 spines μm^−1^ (n = 6 mice) (Figure 1A; Figure S2). Although spines were also detected on basal dendrites (Figure 1B), unambiguous allocation of full basal dendrites to single neurons was not always possible. We thus focused our analysis on apical dendrites. Spines on PVI dendrites were distributed non-homogeneously along the dendritic tree with individual dendritic segments displaying high variability in spine densities ranging from densities reported for DG granule cells (∼1.6 spines μm^−1^ in planar analysis) (Desmond and Levy, 1985) to non-spiny segments (Figure 1C). This non-uniform distribution was pronounced also at the level of the individual segment, where spines formed distinct local clusters with densities of up to 3 spines μm^−1^ (Figure S3).

### Densities of Dendritic Spines Depend on the Source of Afferent Input

According to anatomical and functional criteria, the DG can be divided into several sub-divisions along its longitudinal, transverse, and radial axis (Igarashi et al., 2014, Lee et al., 2014, Shipton et al., 2014, Strange et al., 2014). To probe whether the occurrence of spines in PVIs correlates with anatomical localization, we quantified spine densities at various anatomical subdivisions of the dDG. We found no significant differences in either the percentage of spiny PVIs or the total spine density of PVIs between upper and lower blade, between hemispheres, or along the longitudinal or transverse axis (p > 0.05 for all comparisons; Figure S4). However, we did find that the ratio of spiny versus non-spiny PVIs was higher if somata were located near the hilus compared to the molecular layer (ml) (p = 4.08·10^−7^; one way ANOVA with Bonferroni correction for multiple comparisons; Figure S4).

To investigate how spine formation relates to the source of synaptic inputs, we took advantage of the layered afferent connectivity in the ml of the DG. The inner ml contains intra-hippocampal, commissural, and subcortical projections, whereas the middle and outer mls contain medial and lateral entorhinal cortex projections, respectively (Witter, 2007, Zipp et al., 1989). Sholl analysis of spine densities across the ml revealed striking differences between afferent compartments with a steep decline of mean spine density from the inner to the outer ml (0.29 ± 0.025, inner; 0.09 ± 0.012, middle; 0.01 ± 0.002 spines μm^−1^, outer ml; p < 0.0001 for all comparisons; Figure 1D). This change in spine density across the ml could not be explained by the plain distance from the cell soma (Figure S5), as we found no correlation between soma location and spine density in the ml (Pearson correlation, p = 0.72). These data indicate that spine densities of apical PVI dendrites vary with the source of synaptic inputs.

### Spines of PVIs Participate in the Formation of Synapses

**Figure 2:**
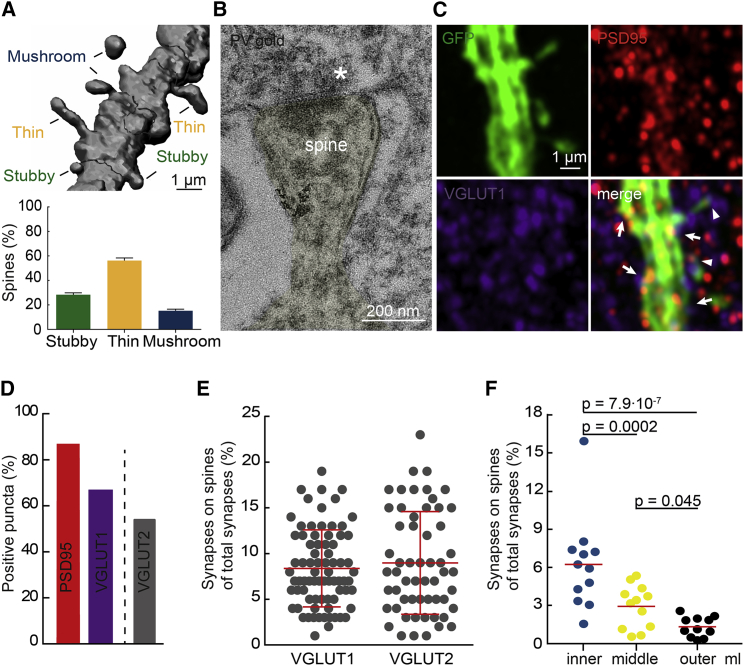
Dendritic Spines Participate in Synapse Formation (A) PVI spines show stubby, thin, and mushroom morphologies. Top, example of a 3D-reconstructed segment. Bottom, thin spines were more abundant than stubby and mushroom spines (n = 5,700 spines, 57 cells, 6 mice). (B) Electron micrograph showing a PV-immunogold-labeled spine (yellow) forming an asymmetrical synapse (asterisk). (C) Single-plane confocal images showing immunoreactivity for GFP (green), PSD95 (red), and VGLUT1 (blue) in the ml. Examples of PSD95-positive puncta in shafts and spines apposed to VGLUT1 positive boutons (arrows and arrowheads, respectively) represent putative synapses. (D) About 90% of all spines were positive for PSD95 and apposed to VGLUT1- or VGLUT2-positive terminals (n = 1,960 spines, 29 neurons, 3 mice). (E) The percentages of VGLUT^+^/PSD95^+^ putative spine synapses over all synapses (shaft and spine) varied in individual spiny segments (n = 134 segments, 29 neurons, 3 mice) and (F) depended on the molecular sublayer when quantified for entire dendritic trees (n = 12 neurons, 3 mice); bars are means, and error bars show SD.

Analysis of spine morphologies using NeuronStudio showed that PVI spines had mushroom, thin, and stubby morphologies in ratios similar to those of hippocampal PCs (Harris et al., 1992) (Figure 2A). Electron microscopy revealed that PV-positive spines formed asymmetrical, putative glutamatergic synapses (Figure 2B). We confirmed this by testing for the presence of excitatory pre- and postsynaptic markers at GFP-labeled PVI spines by immunofluorescence (Figure 2C). We used postsynaptic density protein 95 (PSD-95) as postsynaptic marker and identified presynaptic terminals by labeling for either vesicular glutamate transporter 1 or 2 (VGLUT1 or VGLUT2), which are enriched in cortical and subcortical glutamatergic afferents to the DG, respectively, but also show co-expression within the same terminals (Leranth and Hajszan, 2007). Semi-automated spot analysis on three-dimensional reconstructions of GFP-labeled dendrites using Imaris software (Fogarty et al., 2013) showed that about 90% of all spines were positive for PSD95. PSD95-positive puncta, in turn, were apposed to either VGLUT1- (∼65%) or VGLUT2- (∼54%) positive presynaptic terminals (Figure 2D). As VGLUT1 and 2 can be co-expressed, the percentage of PVI spines participating in synapse formation may lie between 65% and 90%, which is in the range reported previously for interneuron spines (Kawaguchi et al., 2006, Scheuss and Bonhoeffer, 2014). However, the majority of putative synapses onto PVI dendrites was identified not on spines but on shafts. Accordingly, putative synapses on dendritic spines accounted only for a fraction of all putative synapses on PVI dendrites and did not exceed 23% for individual segments (Figure 2E). Quantification of putative synapses for entire dendritic trees showed that these percentages varied significantly with the location in the ml (Figure 2F). In summary, these results indicate that most spines receive putative excitatory synapses, but these synapses present only a fraction of all excitatory synapses made on PVI dendrites.

### Spiny PVIs Show Enhanced Plasticity

**Figure 3:**
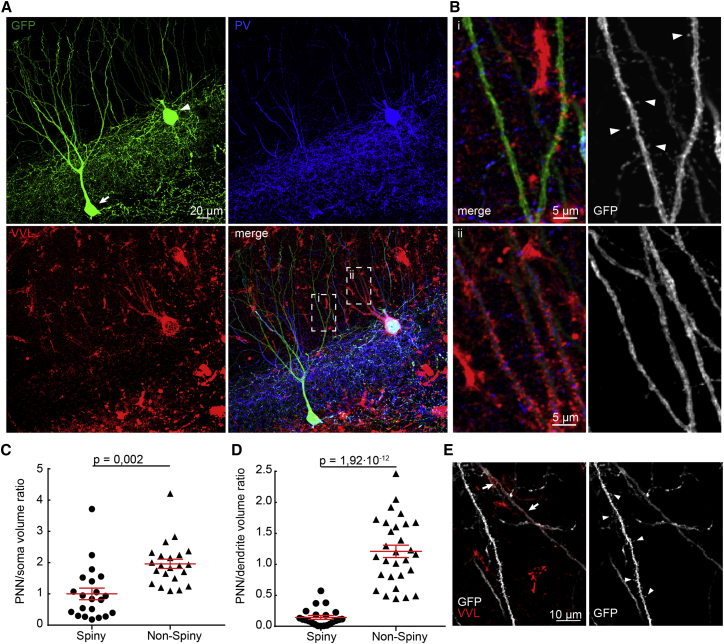
Spiny PVIs and Spiny Segments of PVIs Lack PNNs (A) Confocal image stack showing immunoreactivity for GFP (green) and PV (blue) as well as vicia villosa lectin staining for PNNs (red) for one spiny (arrow) and one non-spiny (arrowhead) PVI. (B) Left, magnifications of the boxed dendrites of the spiny (i) and the non-spiny (ii) PVI in the merged image in (A). Right, corresponding gray scale conversions of the GFP channel. Arrowheads indicate examples of spines. Note that PNNs strongly ensheathe soma and dendrites of the non-spiny neuron but not the spiny neuron. (C and D) Differences in PNN wrapping of somata (C) and dendrites (D) were quantified by calculating the individual volume ratios of the surrounding PNN and the respective soma or dendrite (n = 45 neurons, 5 mice). (E) Example image showing that spiny and non-spiny dendritic segments of the same neuron are differentially enwrapped by PNNs (arrows). Bars are means ± SEM.

**Figure 4:**
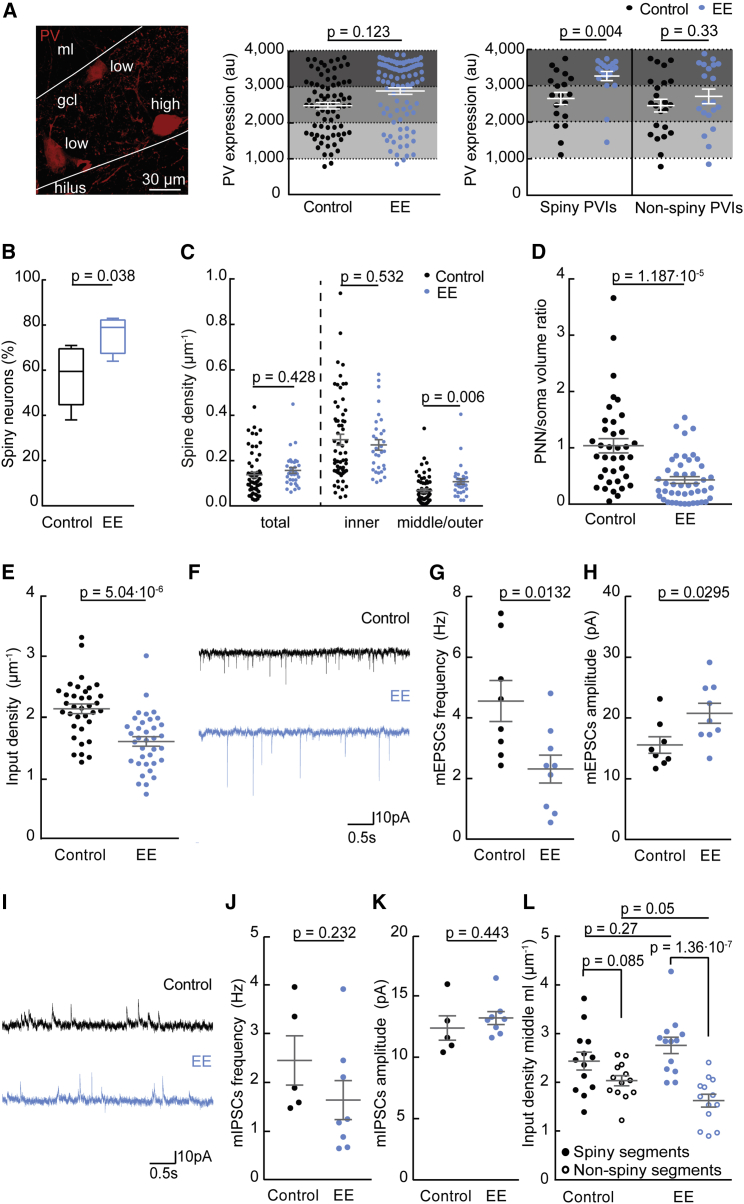
Experience-Induced Plasticity of Spiny PVIs (A) Left, confocal image showing PVIs with different expression levels of PV protein as detected by immunofluorescence (red). Middle, when analyzed for the entire PVI population, PV expression levels were only slightly higher in EE than in control mice (each data point represents PV expression in one cell; n = 168 cells, 3 control and 4 EE mice). Right, however, when analyzed separately these changes in PV expression were significant in spiny but not in non-spiny PVIs (n = 82 cells in 3 control and 4 EE mice). (B) EE exposure increased the proportion of spiny PVIs (n = 144 neurons, 6 control and 4 EE mice). (C) Total spine densities of individual cells as well as spine densities in the inner ml were unchanged after EE, but spine densities in the middle and outer ml were increased (n = 92 cells, 6 control and 4 EE mice). (D) PNN wrapping of PVI somata was strongly reduced after EE compared to control conditions (n = 83 cells in 3 control and 4 EE mice). (E) Total input density (identified as appositions of PSD95- and VGLUT1-positive puncta) of PVIs declined after EE exposure (n = 71 cells in 3 control and 4 EE mice). (F–H) Example traces (F), frequency (G), and amplitude (H) of mEPSCs (n = 8 and 9 cells in 3 control and 4 EE mice) in PVIs of control and EE mice. (I–K) Example traces (I), frequency (J), and amplitude (K) of mIPSCs (n = 5 and 8 cells in 3 control and 4 EE mice) in PVIs of control and EE mice. Note the decrease in frequency but increase in amplitude of mEPSCs. (L) Whereas spiny and non-spiny segments displayed similar input densities (appositions of PSD95- and VGLUT1-positive puncta) in control mice, EE mice showed reduced input densities on non-spiny segments (n = 52 segments in 3 control and 4 EE mice). Bars are means ± SEM.

In PCs, dendritic spines are key sites of plasticity. We thus asked whether spiny PVIs exhibit enhanced adaptive capacities. It is believed that a specialization of the extracellular matrix—the PNN—which ensheathes primarily PVI somata and proximal dendrites, is a key determinant and negative regulator of plasticity for these neurons (Favuzzi et al., 2017, Sorg et al., 2016). In agreement with this concept, we found that PNNs were significantly sparser around somata of spiny compared to non-spiny PVIs (p = 0.002; Figures 3A and 3C). Interestingly, a dramatic difference in PNN wrapping was also evident at the level of dendritic segments, with very weak PNN envelopes around spiny versus non-spiny segments (p = 1.92·10^−12^; Figures 3B, 3D, and 3E). These data indicated that spiny PVIs may indeed be more plastic. To directly test this, we followed recent examples, which have shown that behavioral experience induces plasticity within the hippocampal PVI network, as measured by changes in PV protein expression, which, in turn, correlate with subsequent cognitive performance (Donato et al., 2013). Accordingly, we have used PV expression levels of individual PVIs as a proxy to test whether spiny PVIs and non-spiny PVIs would differ in their responsiveness to behavioral experience. AAV-FLEX-GFP injected mice were either exposed to an enriched environment (EE) for 8 days or held in the home cage for the same time as controls. We then analyzed the intensity of PV-immunoreactivity of individual GFP-tagged PVIs (Figure 4A). In agreement with a recent report (Donato et al., 2015), we found that EE induced a shift toward high PV protein expression, although this shift was not significant (EE versus control; p = 0.123; Figure 4A). However, when we analyzed PV expression levels separately for spiny and non-spiny PVIs, we found a highly significant shift in behaviorally induced PV expression in spiny but not in non-spiny PVIs (p = 0.0042 in spiny versus p = 0.33 in non-spiny PVIs; Figure 4A), suggesting that spiny and non-spiny PVIs differ in their susceptibility to experience-induced changes in gene expression.

### PVI Spines Are Sites of Input-Specific Structural Plasticity

Behavioral experience affects spine morphogenesis in PCs, which, in turn, is thought to be a prerequisite for the long-term storage of memories (Hayashi-Takagi et al., 2015, Holtmaat and Svoboda, 2009, Yuste and Bonhoeffer, 2001). Although spines on interneurons have been shown to undergo structural plasticity (Keck et al., 2011, Pérez-Rando et al., 2017), such plasticity has not been investigated at the level of entire dendritic trees. We thus analyzed the EE and control animals for behaviorally induced spine changes. Whereas EE mice showed an increase in the fraction of spiny PVIs in the dDG compared to control mice (78.25% in EE versus 57.33% in control mice; p = 0.038; Mann-Whitney *U*-test; Figure 4B), there was no change in the spine density of individual PVIs when measured over the entire apical dendritic tree (p = 0.428; Figure 4C). However, we did find sublayer-specific changes in spine densities. Although spine densities in the inner ml were similar between EE and control animals (p = 0.532), EE mice showed higher spine densities than control animals in the input zone of the entorhinal cortex (middle and outer ml) (p = 0.0059; Figure 4C). These structural changes to the PVI network were accompanied by a strong reduction in PNN wrapping of PVI somata (p = 1.187·10^−5^; Figure 4D) and a concomitant increase in the percentage of PVIs with reduced PNN sheathing (91 ± 1 in EE versus 54.33 ± 2.186 in control; p = 1.4·10^−5^; Student’s t test).

In parallel, we investigated whether EE exposure affected the wiring of spiny PVIs. We quantified putative synapses identified as appositions of PSD95- and VGLUT1-positive puncta on dendritic trees of spiny PVIs by using spot analysis software. In agreement with previous reports, we found the density of putative excitatory PVI inputs to be in the range of 2.14 ± 0.079 synapses μm^−1^ in control mice (Donato et al., 2013, Gulyás et al., 1999). In EE mice, however, synapse density was significantly reduced (1.6 ± 0.077 synapses μm^−1^; p = 5.04·10^−6^; Figure 4E), without obvious changes in PSD95 or VGLUT1 puncta size (p > 0.98; n = 32324 and 37462 puncta in 3 control and 4 EE mice, Kolmogorov-Smirnov-Test). Consistent with the reduced synapse density we found the frequency of miniature excitatory postsynaptic currents (mEPSCs) onto DG PVIs to be significantly reduced after EE (p = 0.0132; Student’s t test; Figures 4F and 4G). At the same time, however, the amplitude of mEPSCs was significantly higher in EE versus control mice (p = 0.0295; Student’s t test; Figure 4H), indicating that EE induced not only synaptic pruning but also plastic strengthening of remaining excitatory inputs onto PVIs. In contrast, the frequency and amplitude of miniature inhibitory postsynaptic currents (mIPSCs) did not show obvious changes after EE (p = 0.232 and 0.443, respectively; Student’s t test; Figures 4I–4K). To probe how these changes in excitatory connectivity of PVIs related to the occurrence of spines, we compared changes in putative excitatory inputs between spiny and non-spiny segments in the entorhinal input zone of control and EE mice. Whereas the density of inputs was similar between spiny and non-spiny segments in control animals, EE mice received significantly more inputs on spiny than on non-spiny dendritic segments (control, p = 0.085; EE, p = 1.35·10^−7^; Figure 4L), suggesting that non-spiny segments may be more affected by the pruning of putative excitatory inputs than spiny segments.

## Discussion

In contrast to glutamatergic PCs, mature hippocampal GABAergic interneurons have long been thought to lack dendritic spines. A number of studies have, however, shown that different types of hippocampal GABAergic interneurons do carry spines (Freund and Buzsáki, 1996, Guirado et al., 2014, Gulyás et al., 1992, Scheuss and Bonhoeffer, 2014, Sik et al., 1997, Soriano and Frotscher, 1993) but not PVIs (Freund and Buzsáki, 1996, Sik et al., 1995). The lack of spines has been suggested to be an important factor for rapid signal propagation in PVIs (Hu et al., 2014). Contrary to this assumption, recent studies have reported the sparse existence of spines in neocortical PVIs (Kawaguchi et al., 2006, Sancho and Bloodgood, 2018). We show that within the hippocampal formation different populations of PVIs exist. Whereas PVIs in CA1 and 3 are non-spiny or sparsely spiny, a subpopulation of PVIs in the DG possess considerable numbers of non-homogeneously distributed spines with individual dendritic segments reaching densities previously reported for DG PCs (Desmond and Levy, 1985).

Less than 23% of putative excitatory synapses were formed on dendritic spines, raising the question of how spine synapses may add to information processing in PVIs. A recent study on postsynaptic signaling properties of PVI spines in the mouse visual cortex reported a clear functional division between PVI spine and shaft synapses. Whereas calcium signaling at proximal shaft synapses is modulated by back-propagating action potentials, it is enhanced by activity of neighboring synapses in spine synapses, pointing toward a distinct role of PVI spines in plasticity (Sancho and Bloodgood, 2018). In support of this idea, we found that PNNs, which are known to inhibit plasticity of PVIs, were markedly sparser around spiny PVIs and spiny segments than non-spiny cells and segments (Favuzzi et al., 2017, Sorg et al., 2016). Beyond this, plastic changes in protein expression after EE were induced primarily in spiny but not in non-spiny PVIs, suggesting a functional division between neurons that carry spines and those that do not. However, whether an individual cell is spiny or non-spiny may not be strictly predetermined, as the fraction of spiny neurons was increased after EE alongside a strong reduction in PVI PNN wrapping. To which extent a conversion from non-spiny to spiny PVIs takes place and whether there is indeed a definite non-spiny PVI subpopulation in the DG remain to be investigated. Interestingly, PVIs in CA1 and 3 remained non-spiny (<0.02 spines μm^−1^; n = 16 cells) also after EE, despite documented reductions in the PNN component brevican after EE (Favuzzi et al., 2017).

Experience increased the fraction of spiny PVIs and induced pathway-specific elevations in spine densities. In addition, experience caused reorganization of excitatory inputs onto PVIs, which decreased in density but increased in strength. Interestingly, synaptic pruning differed between spiny and non-spiny dendritic segments. Whereas input densities were similar between spiny and non-spiny segments in control animals, input densities were significantly lower in non-spiny compared to spiny segments in EE mice. These results indicate that experience-driven plasticity is realized not only at the level of entire cells or individual synapses but also at the intermediate scale, which is at the level of dendritic segments with clustered synapses (Kastellakis et al., 2015). Indeed, the organization at the level of dendritic segments is reflected more obviously than in glutamatergic principal neurons by the clustered appearance of spines along the dendritic tree of spiny PVIs (Figures 1C and S3). In light of the particular sensitivity of PVI spine synapses toward the activity of neighboring synaptic input (Sancho and Bloodgood, 2018), we propose that the formation of spine clusters could support the recruitment of PVIs in response to previously encountered associated contextual information from the entorhinal cortex arriving at the dendrite in close spatial proximity (Kastellakis et al., 2015, Takahashi et al., 2012). In this case, electrical interaction between neighboring inputs may enhance calcium signaling at spine synapses to trigger plasticity at co-active inputs (Sancho and Bloodgood, 2018). As PVI dendrites do not support the active backpropagation of action potentials (Sancho and Bloodgood, 2018), which would be required for spike-timing-dependent plasticity at distal dendritic synapses, interactions between clustered inputs may represent an alternative mechanism of plasticity for associated inputs from the entorhinal cortex. Irrespective of our limited knowledge about the cellular and molecular mechanisms underlying spine formation in PVIs, our results point toward a privileged role of these spines and spiny dendritic segments during experience-dependent rewiring of PVIs.

## STAR★Methods

### Key Resources Table

**Table:** 

REAGENT or RESOURCE	SOURCE	IDENTIFIER
**Antibodies**		

Mouse anti-GFP	Invitrogen	Cat# A11120; RRID: AB_221568
Rabbit anti-GFP	Invitrogen	Cat# A6455; RRID: AB_221570
Rabbit anti-PV	Swant	Cat# PV 25; RRID:AB_10000344
Rabbit anti-RFP	Rockland	Cat# 600-401-379; RRID:AB_2209751
Rabbit anti-PSD95	AbCam	Cat# ab18258; RRID:AB_444362
Guinea pig anti-VGLUT1	Millipore	Cat# AB5905; RRID:AB_2301751
Guinea pig anti-VGLUT2	Millipore	Cat# AB2251; RRID:AB_1587626

**Bacterial and Virus Strains**		

AAV 1/2-FLEX-GFP	Murray et al., 2011	N/A

**Chemicals, Peptides, and Recombinant Proteins**		

Isoflurane	Baxter	Cat# FDG9623;
Paraformaldehyde	Sigma-Aldrich	Cat# 441244; CAS: 30525-89-4
Glutaraldehyde 25%	Roth	Cat# 3778.1; CAS: 111-30-8
Triton X-100	Fisher BioReagents	Cat# BP151-500; CAS: 9002-93-1
DAPI	Sigma-Aldrich	Cat# 32670; CAS: 28718-90-3
Mowiol	Sigma-Aldrich	Cat# 81381; CAS: 9002-89-5
SlowFade Gold	Thermo Fisher	Cat# S36937
Vicia villosa lectin	Vector Laboratories	Cat# B-1235; RRID:AB_2336855
Alexa647-conjugated streptavidin	Jackson ImmunoResearch	Cat# 016-600-084; RRID:AB_2341101
Osmium (VIII) oxid	Merck	Cat#: 1245050500 CAS: 20816-12-0
1,2-propylenoxid	Merck	Cat#: 8070270100 CAS: 75-56-9
Araldite M	Sigma-Aldrich	Cat#: 10951
Araldit M accelerator 960	Sigma-Aldrich	Cat#: 10952
Uranylacetate	Merck	Cat#: 108473 CAS: 6159-44-0
Tetrodotoxin citrate	Hello Bio	Cat#: HB1035 CAS: 18660-81-6
QX 314 bromide	Hello Bio	Cat#: HB1029 CAS: 24003-58-5

**Experimental Models: Cell Lines**		

293 [HEK293]	ATCC	CRL-1573

**Experimental Models: Organisms/Strains**		

Mouse: B6;129P2-Pvalb^tm1(cre)Arbr^/J	The Jackson Laboratory	JAX stock #008069
Mouse: B6.Cg-Gt(ROSA)26Sor^tm9(CAG-tdTomato)Hze^/J	The Jackson Laboratory	JAX stock #007909

**Software and Algorithms**		

ZEN 2 (blue edition)	ZEISS	https://www.zeiss.com/microscopy/int/products/microscope-software/zen-lite.html RRID:SCR_013672
LSM 510 version 3.2 SP2	ZEISS	https://www.zeiss.com/microscopy/int/downloads.html
NeuronStudio	Wearne et al., 2005, Rodriguez et al., 2008	https://icahn.mssm.edu RRID:SCR_013798
Imaris 8	Bitplane	https://imaris.oxinst.com/packages
GraphPad Prism 6	GraphPad Software	https://www.graphpad.com/ RRID:SCR_002798
MATLAB R2015a	MathWorks	https://www.mathworks.com/products/matlab.html RRID:SCR_001622
Patchmaster 90.3	Heka	http://www.heka.com/products/products_main.html#soft_pm RRID:SCR_000034
pCLAMP 10.5.2.6 Clampfit	Molecular Devices	https://www.moleculardevices.com/products/software/pclamp.html RRID:SCR_011323

### Lead Contact and Materials Availability

Further information and requests for resources and reagents should be directed to and will be fulfilled by the Lead Contact, Peer Wulff (p.wulff@physiologie.uni-kiel.de).

### Experimental Model and Subject Details

#### Animals

All procedures involving experimental animals were in accordance with the German Animal Welfare Act and approved by the local authorities. PV-Cre mice (Hippenmeyer et al., 2005) were purchased from Jackson laboratories (Repository number 008069) and maintained as heterozygous colonies or crossed with Ai9 Cre reporter mice (Madisen et al., 2010; Jackson laboratories, Repository number 007909). All experimental procedures were carried out on a total of 26 male heterozygous PV-Cre or PV-Cre::Ai9 mice between P38 to P70. Mice were maintained in a 12-h light-dark cycle under standard group housing conditions and were provided with food and water *ad libitum*.

### Method Details

#### Production of recombinant AAV vectors

AAV-FLEX-GFP vectors were produced as described previously (McClure et al., 2011, Murray et al., 2011). Briefly, virions containing a 1:1 ratio type 1 and type 2 capsid proteins were produced by transfecting human embryonic kidney (HEK) 293 cells with the rAAV backbone plasmid pAM-FLEX-GFP along with AAV1 (pH21), AAV2 (pRV1) and adenovirus helper plasmid pFdelta6 using the calcium phosphate method. 48 hours post transfection, cells were harvested and rAAVs were purified using 1 mL HiTrap heparin columns (Sigma) and concentrated using Amicon Ultra centrifugal filter devices (Millipore). Infectious rAAV particles (viral titer) were calculated by serially infecting HEK293 cells stably expressing Cre-recombinase and counting GFP-positive cells.

#### Surgery

Stereotaxic surgeries were carried out as described (Murray et al., 2011). Anesthesia was induced with 3% isoflurane in O_2_ by inhalation and maintained on 1.5%–2% isoflurane throughout surgery. Mouse heads were fixed in a stereotaxic frame (Kopf Instruments, USA), and body temperature was maintained at 37°C using a feedback-controlled heating pad. Analgesic treatment was given locally subcutaneously (Xylonest 2%, AstraZeneca) and intraperitoneally (Rimadyl, 22mg/kg, Zoetis) 5′ before incision. The skull was exposed and small holes were drilled relative to Bregma. Coordinates for dorsal dentate gyrus (DG) injections were AP −1.94 mm, ML ± 1.0 mm, depth −2.1 mm. 1 μl AAV-FLEX-GFP (produced in-house, titer: 6·10^6^ infectious particles per ml) was injected over a ten minute period at each injection site. Injections were made using a Hamilton microliter syringe 701 (Hamilton Company, USA). After injection, the burr holes were filled with bone wax, the skin was replaced and fixed with Vetbond tissue adhesive (3M, USA). Mice were re-hydrated intraperitoneally with Ringer’s solution and monitored following surgery. During the recovery period mice were housed individually.

#### Enriched environment

Behavioral experiments began 10 days after stereotaxic injections. For environmental enrichment mice were group housed with 2 to 3 littermates for 8 days. The enriched cages were 80 cm x 45 cm x 25 cm in size and contained several objects (stairs, tunnels, wooden hanging columns, running wheel, plastic house etc.). These objects were changed or displaced every two days. Age-matched control mice were single housed in standard-sized cages (40 cm x 20 cm x 15 cm) without objects except bedding and nest material. Assignment to experimental groups was random.

#### Histology

Mice were deeply anaesthetized by intraperitoneal injection of Pentobarbital (50 mg per 30 g body weight) and transcardially perfused with phosphate buffered saline (PBS, pH 7.4) for 4 minutes followed by ice-cold 4% paraformaldehyde (PFA) in PBS (plus 1% glutaraldehyde for the electron microscopy) for 10 min. After removal, brains were post-fixed in 4% PFA overnight at 4°C, embedded in 4% agar in PBS and cut into coronal sections on a Leica VT1200S vibratome (thickness 50 μm for quantification of synapses, 80 μm for spine analysis). Free-floating sections were permeabilized in 0.4% Triton X-100 in PBS for 30 min and blocked in PBS containing 4% normal goat serum (NGS) and 0.2% Triton X-100 for 30 min at room temperature. Primary antibodies were diluted in PBS containing 2% NGS and 0.1% Triton X-100 and incubated with sections overnight at 4°C. The following primary antibodies were used: Mouse monoclonal anti-GFP (1:1000, A11120, Invitrogen), rabbit polyclonal anti-GFP (1:1000, A6455, Invitrogen), rabbit polyclonal anti-PV (1:2000, PV25, Swant), rabbit polyclonal anti-RFP (1:2000, 600-401-379, Rockland), rabbit polyclonal anti-PSD95 (1:2000, ab18258, AbCam), guinea pig polyclonal anti-VGLUT1 and anti-VGLUT2 (1:5000, AB5905 and AB2251, Millipore). For labeling of PNNs, we used biotinylated vicia villosa lectin, VVL (B-1235, VECTOR laboratories) at a concentration of 10 μg/ml. Following primary antibody or VVL incubation, sections were washed three times for 10 min in PBS with 1% NGS at room temperature and incubated with secondary antibodies or streptavidin, respectively, for 2 to 3 h at room temperature, protected from light. Secondary antibodies used were: Goat anti-mouse Alexa Fluor 488 (1:1000, A11001, Invitrogen), goat anti-rabbit Alexa Fluor 488 (1:1000, A11008, Invitrogen), goat anti-rabbit Cy3 (1:1000, 111-165-144, Jackson Immunoresearch), goat anti-guinea pig Cy5 (1:500, 106-175-003, Jackson Immunoresearch). For VVL detection Alexa647-conjugated streptavidin was used (1:500, 016-600-084, Jackson Immunoresearch). Sections were then washed once in PBS containing 1% NGS and twice in PBS alone for 10 min. After a quick rinse in distilled water, sections were mounted onto glass slides (Roth), counterstained using DAPI (Sigma) and coverslipped using Mowiol (Sigma) or SlowFade (Thermo Fischer). Brain sections of mice from the environmental enrichment group and the home cage control group were processed in parallel under the same conditions.

#### Imaging

Images were acquired either with a Zeiss Axio-Imager M2 epifluorescent microscope with Apotome, a Zeiss LSM880 confocal laser scanning microscope with Airyscan or a Zeiss LSM510 confocal laser scanning microscope using a Plan-Apochromat 63x oil-immersion objective (numerical aperture (NA) 1.4, Zeiss), a Plan-Neofluar 40x oil-immersion objective with a NA of 1.3 (Zeiss) or a Plan-Neofluar 40x oil-immersion objective with a NA of 1.4 (Zeiss).

For analysis of dendritic spines, 2 to 4 tiles with stacks of 100 to 150 optical slices with an interval of 0.3 μm were captured using a Plan-Apochromat 63x oil-immersion objective (NA 1.4, Zeiss) at the Axio-Imager M2 microscope with Apotome. Scaling per voxel was 0.1 × 0.1 × 0.3 μm. The approximate point-to-point resolution was 0.24 μm. Neurons with largely intact, uninterrupted dendritic trees connected to the soma were chosen. Dendritic sections stretching from one branching point to the next were defined as dendritic segments. Proximal dendrites within the granule cell layer were only analyzed, if interference by axonal processes was low. Otherwise, analysis of proximal dendrites started in the inner molecular layer at the border to the granule cell layer. To investigate percentages of spiny neurons depending on soma location in hilus, gcl or ml (Figure S4), we identified the border of the gcl with the hilus and attributed the somata to one of the three layers according to the distance from this border (within 10 μm, hilus; between 10 and 60 μm, gcl; > 60 μm, ml).

For detection of putative VGLUT1/2+/PSD95+ synapses, slices were imaged with a Zeiss Axio-Imager M2 with Apotome using a Plan-Neofluar 40x oil-immersion objective (NA 1.4 Zeiss; approximate point-to-point resolution was 0.24 μm) or with a Zeiss LSM510 confocal laser scanning microscope using a Plan-Neofluar 40x oil-immersion objective (NA 1.3, Zeiss), zoom 2.4, at an optimal spatial resolution of approximately 0.2 μm in xy and 0.6 μm in z. The z-step size was 0.3 μm. To confirm data obtained at the Apotome and the LSM510 confocal microscope images were also acquired at a LSM880 confocal microscope with Airyscan with a Plan-Neofluar 40x oil-immersion objective (NA 1.4, Zeiss), zoom 2, and a z-step size of 0.18 μm at a very high spatial resolution of approximately 0.12 μm in xy and 0.4 μm in z (see also Software analysis below). For comparison of putative synapses on spines and shafts on individual segments, segments from the inner molecular layer were selected. In contrast, the analysis of putative VGLUT1-positive boutons on PSD95-positive spines and shafts per sublayer was performed on total dendritic trees (including spiny and non-spiny segments) of 12 distinct neurons.

For analysis of vicia villosa lectin stained PNNs and PV labeling intensities, images were taken at a Zeiss LSM510 confocal laser scanning microscope using a Plan-Neofluar 40x oil-immersion objective (NA 1.3, Zeiss). Stacks of 60 to 130 optical slices with a z-step size of 0.3 μm were captured. Settings remained the same during image acquisition for all samples.

#### Software analysis

##### Tracing of dendritic trees and spine detection

For tracing of dendritic trees and spine detection, z stack images were displayed as 2-dimensional maximum intensity projections and analyzed using the semi-automated software NeuronStudio (https://icahn.mssm.edu) as previously described (Rodriguez et al., 2008, Wearne et al., 2005). Largely intact dendrites connected to their soma were traced automatically and manually corrected for accuracy. Individual spines were automatically detected and manually inspected and validated for location and type. Erroneous detections (e.g., short dendritic branches, crossing fibers) and spines with a longitudinal axis parallel to the z axis were manually removed. For spine identification, we used the following default detection parameters: Spine height, 0.2-3 μm; maximum spine width, 3 μm; minimum spine volume, 5 voxels. For spine classification we also used the default parameters. Spines with a head-to-neck diameter ratio greater than 1.1 were classified as thin or mushroom. Spines with a head-to-neck diameter ratio below 1.1 were classified as thin, if the length to head diameter ratio was above 2.5, and as stubby, if the length to head diameter ratio was below 2.5. Spines with a head to neck diameter ratio greater than 1.1 were classified as mushroom, if the head diameter was equal or greater than 0.35 μm. Spine densities were expressed as average number of spines per micrometer along the dendrite’s longitudinal axis.

##### Detection of putative synapses

Putative synaptic contacts on PVI dendrites were analyzed semi-automatically according to published protocols (Belmer et al., 2017, Fogarty et al., 2013). Image stacks showing immunoreactivity for GFP, PSD95 and VGLUT1 or VGLUT2 were used for three-dimensional reconstructions using Imaris software (Bitplane AG). A solid surface best matching the dendritic anatomy was generated using the “surface” tool. The background was subtracted. The “smoothing” tool was disabled, to avoid artificial uniformity. GFP voxel histograms were used to determine the threshold for background exclusion. Channels for pre- and postsynaptic markers were filtered based on spatial relationship to the neuronal surface (removal of presynaptic fluorescent signals within the surface, and postsynaptic fluorescent signals outside the surface). For quantification of pre- and postsynaptic puncta the diameter as measured in “slice view” mode was set between 0.5 and 0.9 μm for PSD95 (labeling the PSD of excitatory synapses), and 0.6 and 1.1 μm for VGLUT1 or VGLUT2 (labeling the synaptic vesicle zone of excitatory presynaptic terminals), corresponding to reported size ranges (Belmer et al., 2017, Fogarty et al., 2013, Kim and Sheng, 2009, Kitahara et al., 2016). The minimum size threshold was chosen higher than the z-step interval to ensure detection in more than one optical section to avoid false positive detection (Belmer et al., 2017, Fogarty et al., 2013). Pre- and postsynaptic puncta within the set size range were detected using the Imaris spot function to create discrete spheres with coordinates for the center of mass. Putative synapses were detected by quantification of pre- and postsynaptic spheres opposed to each other with a maximum distance of 1 μm (the sum of the maximi radii of puncta) according to published protocols (Fogarty et al., 2013, Klenowski et al., 2015). Fidelity of synapse detection was confirmed by moving the channel reporting immunoreactivity of VGLUT1 or VGLUT2 against the channels reporting immunoreactivity of GFP and PSD95. On a sample of 18 neurons either mirroring (along the x, y or z axis) or shifting (along the x axis) of the channels resulted in a strong reduction of detected putative synapses by about 35 to 45% (p < 0.0001 in all multiple comparisons; one way ANOVA with Bonferroni correction).

Data obtained from Apotome and LSM510 confocal images were confirmed by data obtained from high resolution images acquired at the LSM880 with Airyscan (see above) by comparing synapse densities for a sample of PVI dendritic segments, which were similar for the three microscopes (2.75 ± 0.28 versus 2.85 ± 0.35 versus 2.9 ± 0.3 synapses μm^-1^ at Apotome, LSM510 and LSM880, respectively; p = 0.91; one way ANOVA with Bonferroni correction for multiple comparisons; 5 segments at each microscope).

For analysis of PSD95- and VGLUT1-positive puncta sizes we used the spot growing function in Imaris (Imaris 8.0.0, BitplaneAG), selecting the functions “local contrast” and “region growing diameter from border” to obtain a cumulative distribution of puncta sizes between 0.1 and 1.6 μm in 0.1 μm bins.

#### Quantification of PNN volumes

For analysis of PNN volumes around somata, a three-dimensional isosurface was generated for each intact GFP-labeled soma using Imaris software (Imaris 8.0.0, BitplaneAG). An additional three-dimensional isosurface was then generated for the VVL-positive PNNs surrounding the soma plus the proximal 25μm of its apical dendrite. We then calculated the PNN to soma volume ratio for each neuron. For analysis of PNN volumes around dendrites, we used a similar approach by generating three-dimensional isosurfaces for 25 μm long stretches of GFP-positive dendrites in the inner ml and their surrounding VVL-positive PNN volume and calculation of the PNN to dendrite volume ratio. To compare percentages of PVIs with low PNN sheathing in EE and control mice, we set an upper PNN/soma volume ratio threshold of 1 (the mean PNN/soma volume ratio in control mice).

##### Quantification of PV labeling intensity

Analysis of PV expression levels was carried out for somata completely integrated in the tissue section as described previously (Donato et al., 2013). Three-dimensional isosurfaces were generated for each labeled soma (number of voxels > 1). PV immuno-fluorescence intensities were then quantified automatically as a mean of all voxels in arbitrary units (Imaris 8.0.0, BitplaneAG). The range and internal boundaries of fluorescent intensity values were defined by dividing the range of intensity values obtained into four symmetrical sections: low 0-1000 arbitrary confocal units (au), intermediate low 1000-2000 au, intermediate high 2000-3000 au, high 3000-4000 au.

#### Pre-embedding immuno-electron microscopy

Embedding and sectioning of tissue samples was performed as described previously (Schneppenheim et al., 2017). Mouse monoclonal anti-PV (1:1000, P3088 Sigma) antibodies were covalently conjugated to 20nm gold particles (GOLD conjugation kit, abcam, ab188215). Immunostaining procedures were as described above. After a final wash samples were stored in 3% Glutaraldehyde prior to post-fixation in 2% osmium-tetroxide for 2 h. After dehydration in an ascending row of ethanol (50%–100%) samples were treated with 1,2-propylenoxid for 2x 10 min. Araldit + 3% enhancer (mixed 1:1 with 1,2-propylenoxid) was added over night at room temperature, exchanged to Araldit + 2% enhancer for 5 h at room temperature and exchanged again to Araldit + 2% enhancer at 60°C for 48 h for polymerization. From the samples semi-thin (0.5 μm) and thin (60 nm) sections were prepared at different levels and stained with uranyl acetate and lead citrate. Using the semi-thin sections enabled to identify the dentate gyrus and simplified orientation. The corresponding thin section was then transferred into a transmission electron microscope (JEM1400 Plus, JEOL, Germany) operating at 100 kV acceleration voltage and the area of the dentate gyrus was imaged. Images were captured with a 4kx4k digital camera (F416, TVIPS, Germany).

#### Electro-physiology

##### Slice preparation

PV-Cre::Ai9 mice were anesthetized with isofluorane and transcardially perfused with an ice-cold cutting solution containing (in mM): NaH_2_PO_4_ 1.25, KCl 2.5, MgSO_4_ 10, CaCl_2_ 0.5, N-Methyl-D-glucamine (NMDG) 92, Glucose 25, NaHCO_3_ 30, HEPES 20, Na Ascorbate 5, Na Pyruvate 4. Transversal slices of 350 μm from the dorsal hippocampus were cut in the same ice cold and oxygenated solution on a Thermo Scientific HM650V vibratome. Slices were first transferred to an incubation chamber containing the same solution heated to 35°C for 12 min. Slices were then transferred into a storing chamber at room temperature filled with a HEPES-based solution containing (in mM): NaH_2_PO_4_ 1.25, KCl 2.5, MgSO_4_ 2, CaCl_2_ 2, NaCl 92, Glucose 25, NaHCO_3_ 30, HEPES 20, Na Ascorbate 5, Na Pyruvate 4, and kept there for at least 30 min before recordings.

##### Patch clamp recordings and analysis

Slices were transferred to a recording chamber superfused at 3ml/min with ACSF at room temperature containing (in mM): NaH_2_PO_4_ 1.25, KCl 2.5, MgSO_4_ 2, CaCl2 2, NaCl 119, Glucose 12.5, NaHCO_3_ 24, Na Ascorbate 5, Na Pyruvate 3. PV interneurons were identified at an Olympus 3X51WI microscope, with fluorescence illumination (585 nm - Cool Led, pE Excitation System) and infrared-differential interference optics (Hamamatsu) through a 20x/1.00 NA water-immersion objective (XLUMPLFLN-W, Olympus). Patch microelectrodes (3–6 MΩ) were pulled from borosilicate glass (1.05 × 1.5 × 100mm GB150TF-10, Science Products) using a horizontal puller (model P-1000, Sutter Instruments). Voltage-clamp (mEPSCs and mIPSCs) recordings were performed using an intracellular solution containing (in mM): CsMetSO_4_ 125, MgCl_2_ 2, CsCl 2, MgATP 4, EGTA 0.5, Na_2_GTP 0.3, Na_2_Phosphocreatin 10, HEPES 10, QX314-Br 5, pH 7.3-7.35 and Biocytin 0.3%. mEPSC were recorded at a holding potential of −70 mV and mIPSCs at 0mV with a HEKA amplifier. For mEPSC and mIPSC recordings, 1 μM TTX was added to the bath and no other pharmacological antagonists were used in order to allow recordings of mEPSCs and mIPSCs from the same cell. Data were filtered on-line at 3 kHz, and acquired at a 20 kHz sampling rate using Patchmaster software (HEKA). After patch clamp recordings, slices were fixed in PFA 4% overnight at 4°C and PV expression was subsequently verified with immunofluorescence as described above. Synaptic currents were analyzed semi-automatically with ClampFit (10.5.2.6, Molecular Devices), using detection parameters of 7pA for event threshold.

### Quantification and Statistical Analysis

For analysis of dendritic spines all neurons within a section were chosen, which showed largely intact, uninterrupted dendritic trees connected to the soma. For quantification of volumes, fluorescence intensity and putative synapses, all neurons within a section were chosen, but those immediately adjacent to the surface were excluded from analysis to avoid artifacts due to reconstruction inaccuracy and saturated staining. For experiments involving enriched environments, age matched mice were randomly assigned to home cage or enriched groups. Mice from enriched environment and controls were analyzed blindly for PV expression levels, PNN wrapping, density of putative synapses and mEPSCs/mIPSCs. Percentages of spiny PVIs and spine densities were initially analyzed un-blinded, but were validated by a blinded independent second experimenter. Sample sizes are given in the figure legends or text. “n” represents either mice, neurons, segments or synapses as indicated. Statistical analysis was performed using MATLAB and GraphPad Prism version 6. When n represented multiple neurons or segments from a relatively low number of subjects, we accounted for intra-class correlation due to clustered data in form of random effects. Accordingly, unless stated otherwise in the text, we applied a linear mixed-effects model using MATLAB (y = Xβ+Zu+ε, where y is the response variable, X is the predictor variable, β is a vector of the fixed-effects regression coefficients, Z is the random effect variable, u is a vector of the random effects and ε is a vector of the residuals). This approach maintains information about variability and avoids under-estimations of the p value (Wilson et al., 2017). We also used Mann-Whitney *U*-test, one way ANOVA with Bonferroni correction for multiple comparisons, Kolmogorov-Smirnov-Test and two-tailed unpaired Student’s t test when appropriate. For correlation analysis, Pearson product moment correlation coefficient was used. A p value of less than 0.05 was considered significant. Unless stated otherwise, all data are shown as mean ± SEM. In figures bars represent means, bars with error bars refer to means ± SEMs. Circles and dots represent individual data points.

## Supplemental Information

Document S1. Figures S1–S5

Document S2. Article plus Supplemental Information
