# Supplementary material for: Spiny and Non-spiny Parvalbumin-Positive Hippocampal Interneurons Show Different Plastic Properties
Source: Cell Rep. 2019 Jun 25;27(13):3725–3732.e5. doi: 10.1016/j.celrep.2019.05.098 (PMC6613043; doi:10.1016/j.celrep.2019.05.098)
Supplement: Document S1. Figures S1–S5 [file mmc1.pdf]

Cell Reports, Volume *27*

Supplemental Information

Spiny and Non-spiny Parvalbumin-Positive Hippocampal Interneurons Show Different Plastic Properties

Angelica Foggetti, Gilda Baccini, Philipp Arnold, Thomas Schiffelholz, and Peer Wulff

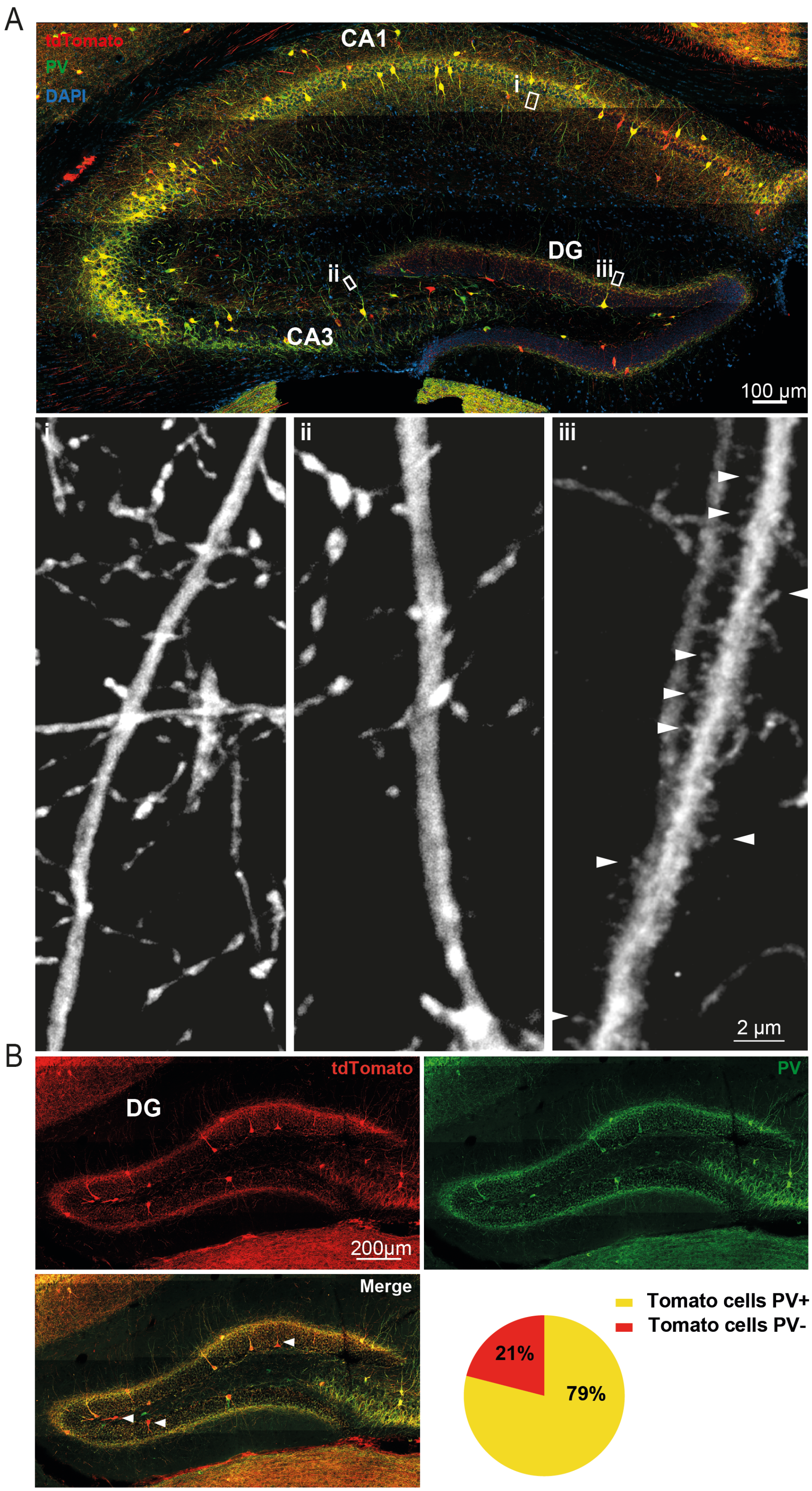

A
tdTomato
PV
DAPI
CA1
i
DG
ii
iii
CA3
100 µm
i
ii
iii
2 µm
B
tdTomato
DG
200µm
PV
Merge
Tomato cells PV+
Tomato cells PV-
21%
79%

Figure S1. tdTomato-labeled PVIs in the dentate gyrus (DG), but not in cornu ammonis (CA) regions 1 and 3 carry dendritic spines (related to Figure 1). (A) *Top*, Apotome image-stack of the hippocampus of a PV-Cre::Ai9 mouse showing immunoreactivity for tdTomato (red), parvalbumin (PV, green) and DAPI (blue). *Bottom*, grey-scale magnifications of boxed areas from CA1 (i), CA3 (ii) and DG (iii) visualizing dendrites of PV-immunoreactive, tdTomato-positive neurons. Note high spine densities in the DG but not CA1 or 3. Arrowheads indicate examples of spines. (B) Apotome image stack of the DG showing immunoreactivity for tdTomato (red) and PV (green). About 80% of tdTomato-positive cells were immunoreactive for PV in the DG (n=255 neurons in 3 mice). Arrowheads indicate tdTomato-positive but PV-negative neurons. Images in the top panel of (A) and panel (B) were created by stitching together high magnification image tiles using ZEN 2 software (Zeiss).

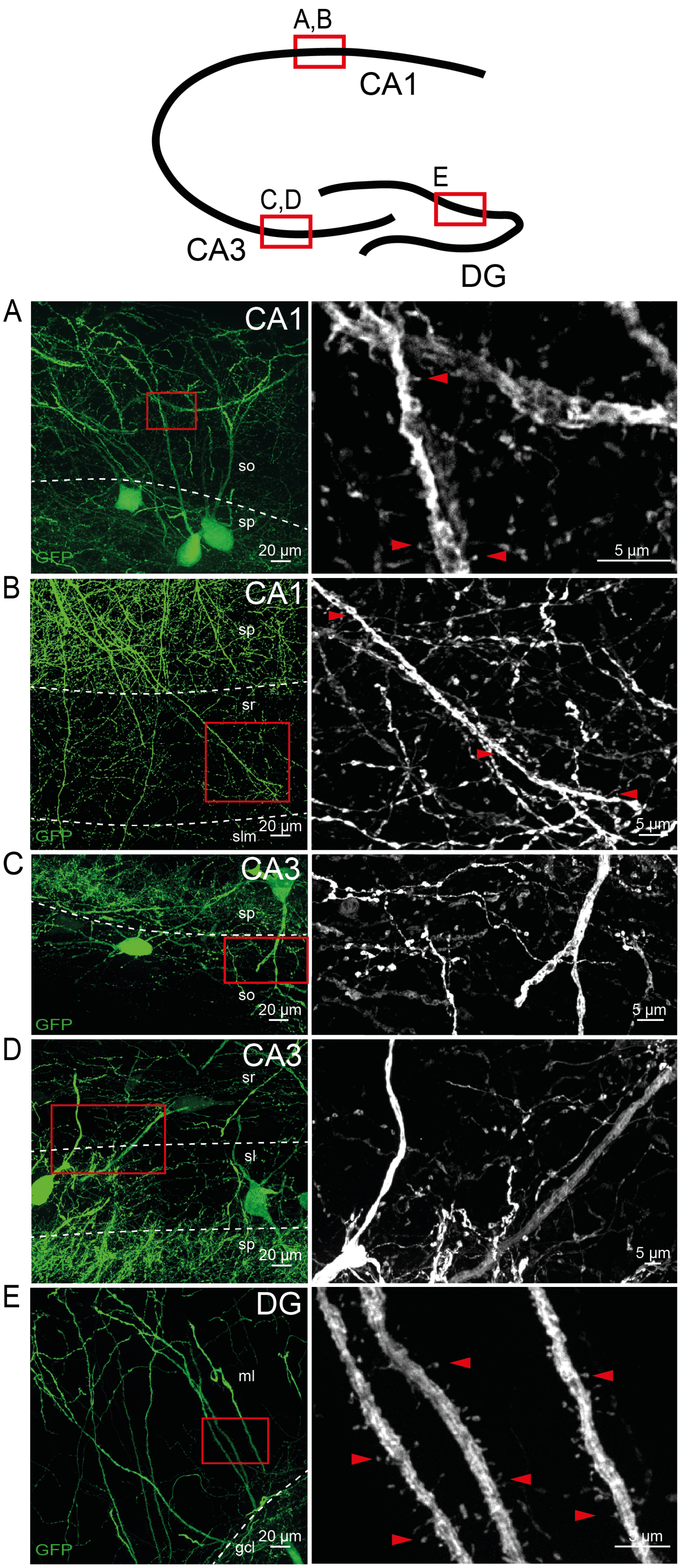

A,B
CA1
C,D
E
CA3
DG
A
CA1
so
sp
GFP
20 µm
5 µm
B
CA1
sp
sr
slm
GFP
20 µm
5 µm
C
CA3
sp
so
GFP
20 µm
5 µm
D
CA3
sr
sl
sp
20 µm
5 µm
E
DG
ml
gcl
GFP
20 µm

Figure S2. After stereotactic injection of AAV-FLEX-GFP into different regions of the hippocampus, GFP-labeled PVIs with high spine densities were detected in the DG but not in CA regions (related to Figure 1). (A-E) Examples of virus-mediated expression of GFP in PVIs of CA1 (A,B), showing basal and apical dendrites, respectively; (C) CA3b; (D) CA3c and (E) DG. The right panels show grey-scale converted magnifications of boxed areas from left panels. Red arrowheads indicate examples of spines. gcl, granule cell layer; ml, molecular layer; sl, stratum lucidum; slm, stratum lacunosum-moleculare; so, stratum oriens; sp, stratum pyramidale; sr, stratum radiatum. Images in (C-E) were created by stitching together high magnification image tiles using ZEN 2 software (Zeiss).

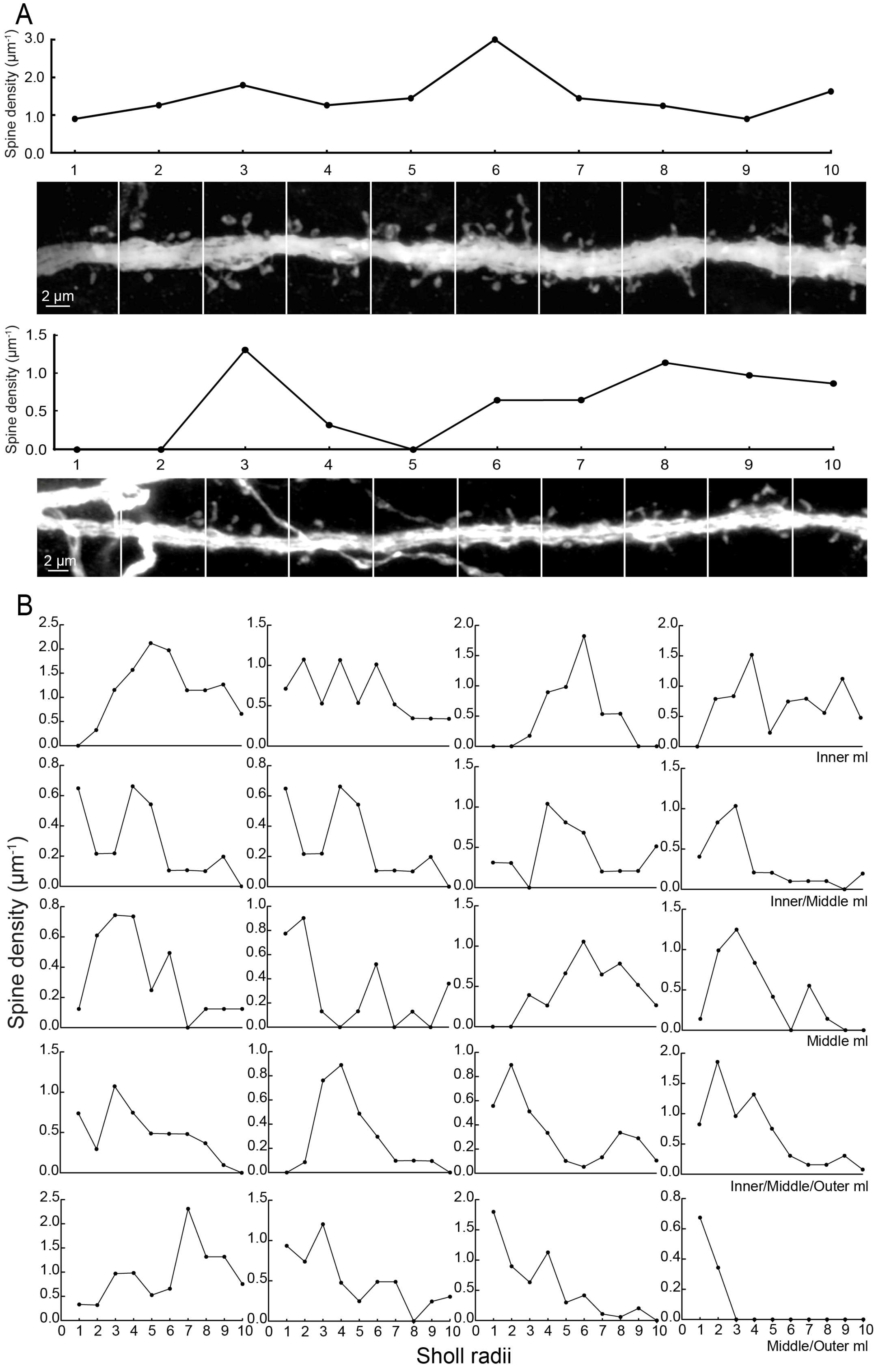
A
Spine density (µm⁻¹)
3.0
2.0
1.0
0.0
1 2 3 4 5 6 7 8 9 10
2 µm
1.5
1.0
0.5
0.0
B
Inner ml
Inner/Middle ml
Middle ml
Inner/Middle/Outer ml
Middle/Outer ml
Spine density (µm⁻¹)
Sholl radii
0 1 2 3 4 5 6 7 8 9 10

Figure S3. PVI spines do not show homogeneous distributions but form clusters (related to Figure 1). (A) Examples of grey-scale converted confocal images of GFP-labeled dendritic segments from the ml of the DG. Vertical white lines indicate Sholl radii (10 per dendritic segment), generated using NeuronStudio. Graphs above confocal images show quantifications of spine densities for the corresponding radii. (B) Quantification of spine densities per Sholl radius for 20 dendritic segments from different sublayers of the DG ml. Note the pronounced variation in spine densities along single segments.

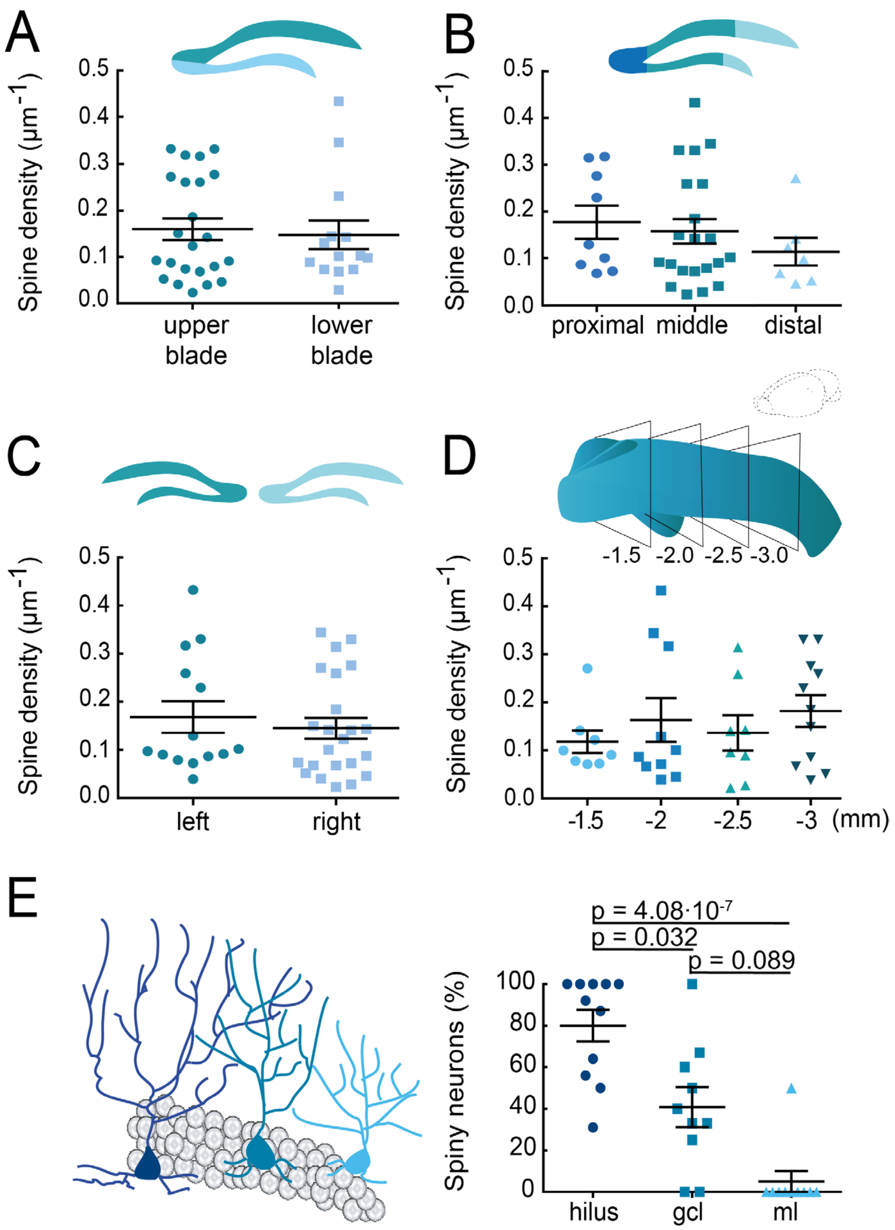

Figure S4. Total spine densities of apical dendrites and fractions of spiny PVIs in relation to the anatomical location of the soma (related to Figure 1). (A-D) In the dorsal DG, spine density was similar for PVIs located (A) in the infra- and suprapyramidal blades, (B) at different transverse positions, (C) in the left or right hemisphere, and (D) at different positions along the rostro-caudal axis of the dDG (n=37 neurons, 3 mice, $p > 0.05$ for all comparisons). (E) *Left*, illustration of PVIs with somata at the hilus-gcl border, within the gcl and at the gcl-ml border. *Right*, analysis of 87 neurons near the hilus, 41 in the gcl and 20 near the ml, showed that the percentage of spiny PVIs was significantly higher for neurons with a soma location near the hilus compared to those with a soma in the gcl or near the ml (n=11 mice). Bars show means; error bars represent SEM.

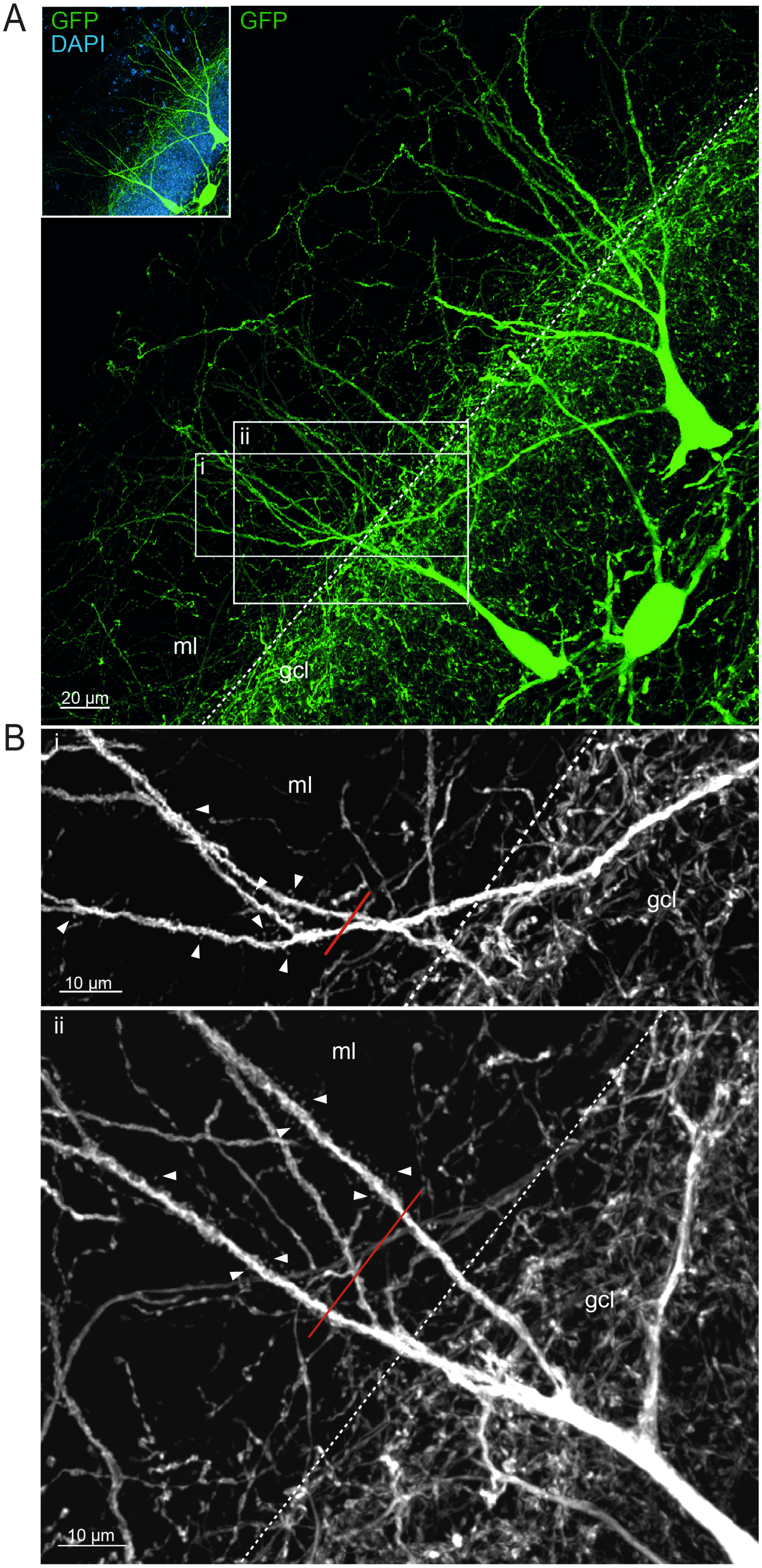
A
GFP
DAPI
GFP
ii
i
ml
gcl
20 µm
B
i
ml
gcl
10 µm
ii
ml
gcl
10 µm

Figure S5. Dendritic spine densities of PVIs depend on the source of synaptic inputs (related to Figure 1). (A) Confocal image stack of GFP+ PVIs with dendrites extending through the gcl into the ml. The DAPI stain in the inset marks the gcl, the dotted line indicates the gcl-ml border. (B) Grey-scale converted magnifications of boxed areas in A. Note that the dendrites are largely non-spiny as they pass through the gcl. However, as the dendrites reach the ml there is an abrupt increase in spine densities (red line), suggesting that spine densities vary with the input not with the distance from the soma. Arrowheads indicate examples of spines. The image in the inset (A) was created by stitching together high magnification image tiles using ZEN 2 software (Zeiss).
